# Metabolic response patterns in brain microdialysis fluids and serum during interstitial cisplatin treatment of high-grade glioma

**DOI:** 10.1038/s41416-019-0652-x

**Published:** 2019-12-10

**Authors:** Benny Björkblom, Pär Jonsson, Pedram Tabatabaei, Per Bergström, Mikael Johansson, Thomas Asklund, A. Tommy Bergenheim, Henrik Antti

**Affiliations:** 1 Department of Chemistry, Umeå University, Umeå, Sweden; 2 Department of Clinical Neuroscience, Neurosurgery, Umeå University, Umeå, Sweden; 3 Department of Radiation Sciences, Oncology, Umeå University, Umeå, Sweden

**Keywords:** Cancer metabolism, Prognostic markers, Cancer therapy

## Abstract

**Background:**

High-grade gliomas are associated with poor prognosis. Tumour heterogeneity and invasiveness create challenges for effective treatment and use of systemically administrated drugs. Furthermore, lack of functional predictive response-assays based on drug efficacy complicates evaluation of early treatment responses.

**Methods:**

We used microdialysis to deliver cisplatin into the tumour and to monitor levels of metabolic compounds present in the tumour and non-malignant brain tissue adjacent to tumour, before and during treatment. In parallel, we collected serum samples and used multivariate statistics to analyse the metabolic effects.

**Results:**

We found distinct metabolic patterns in the extracellular fluids from tumour compared to non-malignant brain tissue, including high concentrations of a wide range of amino acids, amino acid derivatives and reduced levels of monosaccharides and purine nucleosides. We found that locoregional cisplatin delivery had a strong metabolic effect at the tumour site, resulting in substantial release of glutamic acid, phosphate, and spermidine and a reduction of cysteine levels. In addition, patients with long-time survival displayed different treatment response patterns in both tumour and serum. Longer survival was associated with low tumour levels of lactic acid, glyceric acid, ketoses, creatinine and cysteine. Patients with longer survival displayed lower serum levels of ketohexoses, fatty acid methyl esters, glycerol-3-phosphate and alpha-tocopherol, while elevated phosphate levels were seen in both tumour and serum during treatment.

**Conclusion:**

We highlight distinct metabolic patterns associated with high-grade tumour metabolism, and responses to cytotoxic cisplatin treatment.

## Introduction

Despite trimodal treatment based on surgery, radio-chemotherapy, and adjuvant chemotherapy, patients with glioblastoma, the most common primary brain tumour, have a median survival of only 14 months.^1^ This poor prognosis is due to many obstacles in the treatment. Parts of the tumour, especially the invasive migrating tumour cells outside the solid tumour areas, are often behind the blood–brain barrier, which makes it difficult for many systemically administered cytotoxic drugs to penetrate the tissue.^2^ Inside the tumour the blood–tumour barrier is partly disrupted, but still penetration of systemically administered drugs can be inefficient. The blood–brain and the blood–tumour barriers contribute to the fact that very few new cytotoxic drugs or targeted therapies have been successful in clinical trials. Another issue is the lack of tools for early evaluation of the effect of any given treatment. Today, magnetic resonance imaging (MRI) is used to determine changes in tumour size, the most important criterion for response evaluation. However, to improve response monitoring new functional predictive response-assays based on cellular effects are needed. Functional MRI, including MR spectroscopy, could provide a feasible technique for assessing early treatment response.^3,4^ However, the number of detectable metabolites using MR spectroscopy is still low and limited by the sometimes small or even non-visible postoperative tissue volume. Another challenge is pseudo-progression, which makes imaging unreliable during the first 12 weeks after radiotherapy.^4–7^

Cisplatin exerts a strong cytotoxic effect on glioma cells.^8–10^ However, due to the limited penetration of the blood–brain barrier it is difficult to administer cisplatin systemically in relevant doses without dose-limiting neuro- or nephrotoxicity. Therefore, interstitial drug administration is preferable.^11^ In the present study, we have circumvented the blood–brain barrier by delivering cisplatin locally into the tumour tissue using retrograde microdialysis.^12,13^ In addition, microdialysis allowed us to measure changes in the metabolome in the dialysate from the extracellular space in tumour tissue and brain tissue adjacent to tumour (BAT). We used mass spectrometry methods combined with chemometric multivariate statistical analysis^14^ to detect, early metabolic marker or a metabolic pattern in tumour, BAT and serum, and to find possible biomarkers for predicting treatment responses.

## Materials and methods

### Patients

Nine patients with histologically verified glioblastoma and one with anaplastic oligoastrocytoma (pat no. 10) were included in the phase 1 trial, after tumour recurrence following second line chemotherapy. There were five male and five female patients with a mean age of 54.3 (range 40–80) years. Two patients have had several surgeries and a secondary malignification of their tumour from grade II to IV (no. 5) and from grade II to III (no. 10). Nine patients had been operated on by total or sub-total resection and one with biopsy only (no. 2). Resected tumours from patient no. 3 and no. 10 were confirmed to carry a mutation in the gene encoding isocitrate dehydrogenase 1 (IDH1, R132H). All patients were treated primarily with radiotherapy and concomitant temozolomide followed by up to six cycles of adjuvant temozolomide. At recurrence, all patients, except nr. 6 and 7, have also been given second line treatment with bevacizumab based chemotherapy.^15^ At further progression of the disease, the patients were found not suitable for further resection or any other conventional chemotherapy. When entering the study, the patients had an estimated survival of ~3 months. After interstitial treatment using microdialysis, two patients with long survival did, after further tumour progression, receive irinotecan/bevacizumab (pat. no. 4, 10 months after cisplatin) and reirradiation 3.4 Gy X 10 (pat. no. 10, 7 months after cisplatin), respectively.

### Surgery

Stereotactic implantation of microdialysis catheters was performed under general anaesthesia and with the aid of a Leksell stereotactic frame (Elekta, Stockholm, Sweden). A stereotactic contrast-enhanced computerised tomography (CT) was performed for target calculations. The stereotactic method to introduce the microdialysis catheters has been previously described in detail.^16^ The number of catheters used were based on the volume of the tumour tissue and previous experience calculating that the drug could penetrate approximately 10 mm from the catheter.^12^ One catheter was placed in BAT ~10 mm outside of the contrast enhancing lesion. In the first three patients, the intracranial catheters used were Dyphylon Cardiac Catheter (100 kDa), in the fourth patient CMA 62 (100 kDa), and the remaining six patients CMA 71 (20 kDa). Another catheter, CMA 71 (100 kDa) in the first three patients and CMA 60 (20 kDa) in the following, was placed as systemic reference in the abdominal subcutaneous tissue. All catheters had similar semipermeable polyamide membrane from the same manufacturer.

### Treatment—microdialysis

The first night after surgery the patients recovered at the neuro-intensive care unit and baseline microdialysis samples were collected. The following day, the patients were mobilised, and administration of cisplatin was started in one of the tumour catheters, i.e. the most centrally located. On day 2 after surgery administration was started in the remaining tumour catheters. This procedure allowed us to study the pharmacokinetics of cisplatin in the tumour tissue.

The study was designed as a phase 1 study with toxicity as the primary objective. The study protocol was based on a stepwise dose-escalation giving the first four patients a dose of 1 mg/day for a period of 12 days. The following patients were planned to be given 3 or 5 mg/day. Cisplatin was used in a solution of 1 ml containing 1 mg cisplatin in 9 mg/ml NaCl (Cisplatin Meda® (pat 1–5), Cisplatin Ebewe® (pat 6), Cisplatin Accord® (pat 7–9), or Cisplatin Hospira® (pat 10)). The solution used for patient no. 10 contained 1 mg/ml mannitol, added by the manufacturer.

The concentration of cisplatin in the dialytic fluid was based on in vitro experiments and the flow-rate in each catheter was decided depending on the aimed daily dose and the number of catheters used. The catheters were connected to a 2.5 mL syringe placed in a micro infusion pump with an adjustable flow rate of 0.1–5.0 μL/min (CMA 107; CMA Microdialysis). All catheters were perfused with cisplatin 1 mg/ml or, before start of treatment and in the reference catheter, with an isotone Ringer solution (Perfusion fluid CNS; CMA Microdialysis). The pumps were attached to the head dressing of the patients and allowed the patients to move freely at the ward.

**Fig. 1:**
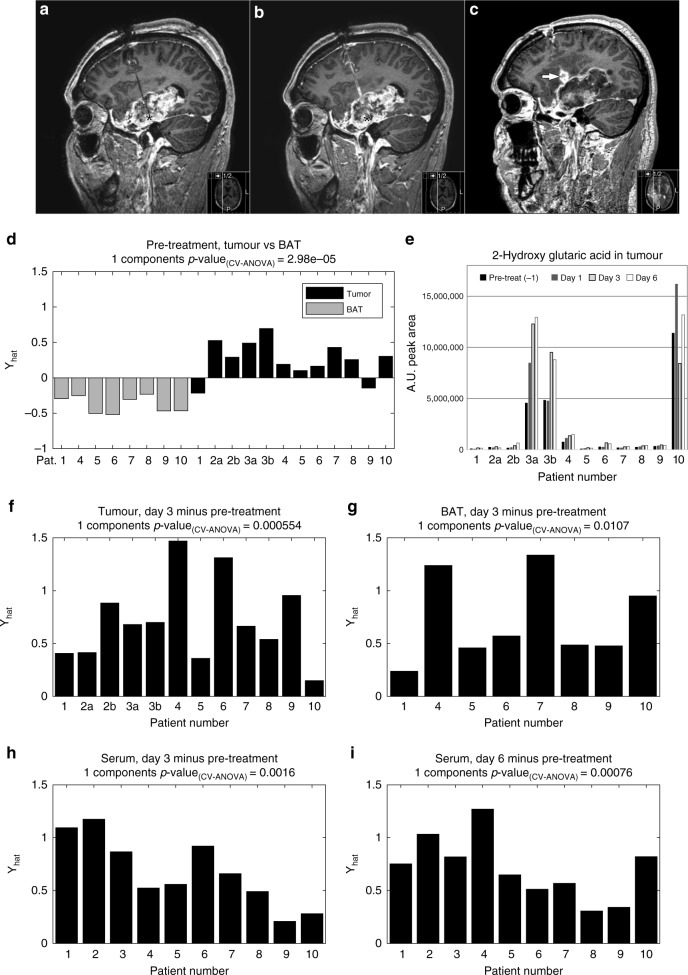
Contrast-enhanced MR-images of patient no. 5. **a** Before start of treatment. **b** After five days of cisplatin treatment. Black asterisk in A–B marks tip of microdialysis catheter. **c** One months after ten days of cisplatin treatment demonstrating widespread necrosis in the tumour area. White arrow in C marks inflammatory response at the catheter site. **d** Multivariate statistical OPLS-DA model comparing metabolite levels detected in microdialysis fluids from tumour and BAT. **e** Relative quantification of 2-hydroxy glutaric acid concentration in microdialysis fluids from the extracellular compartment of tumour tissue pre-treatment, and after one to 6 days of cisplatin treatment. For patient number 2 and 3, dialysate from two treatment catheters was available for analysis, denoted 2a, 2b, 3a and 3b. **f**, **g** Paired multivariate OPLS-EP models comparing metabolite levels detected in microdialysis fluids from tumour (**f**) and BAT (**g**) prior to treatment to levels after 3 days of cisplatin treatment. **h**, **i** Paired multivariate OPLS-EP models comparing metabolite levels detected in serum prior to treatment to levels after 3 days (**h**) or 6 days (**i**) of cisplatin treatment. Statistical significances for the cross-validated models are indicated by CV-ANOVA.

The patients were carefully monitored, and blood samples were obtained during the treatment to evaluate renal or haematological toxicity and to provide pharmacokinetic information. Routine blood samples, blood cell count, electrolytes, liver enzymes and c-reactive protein were analysed preoperatively, day 1, 3, 6 and 12 and also when clinically needed. The concentration of cisplatin in blood was also monitored to evaluate if any cisplatin entering the circulation. All patients performed a CT-scan and an MRI preoperatively (Fig. 1a–c). To confirm the location of the catheters a postoperative CT was performed before the treatment was started. MRI investigations were performed during and immediately after treatment. The patients were followed-up at 1 month after treatment and thereafter every 3rd months until death.

### Sampling

The microdialysis samples were collected every second hour and stored in a refrigerator (temperature 8 °C) until transferred to a −80 °C freezer every 12 h. Five timepoints were selected for analysis of the microdialysis samples. These samples were fasting samples collected between 4 am and 6 am the day before treatment, 1, 3 and 6 days into treatment and after the treatment has ended. Fasting serum samples were collected in plain tubes in the morning before start of treatment, 3 and 6 days into the treatment, and after the end of treatment. Serum samples were immediately frozen at −80 °C following centrifugation.

### Metabolomic analysis of microdialysate and serum

#### Metabolite extraction

The samples were randomly divided into analytical batches, preserving individual patient sample groups and sampling source (tumour, BAT, serum). Frozen 50 µl aliquots of microdialysis liquid and serum were thawed on ice at room temperature. Metabolite extraction was performed using 450 µl methanol:water extraction mix (90:10 v/v, including internal standards (6.75 ng/μl), followed by rigorous agitation at 30 Hz for 2 min in a bead mill (Retsch, MM 400)). The samples were incubated on ice for 2 h and centrifuged at 18,600 × g for 10 min at 4 °C. After pre-clearing, 200 μl supernatant were transferred to GC vials and evaporated until dry in a speedvac. Dried samples were methoxyaminated by the addition of 15 μl methoxyamine in pyridine (15 μg/μl), shaken for 10 min at room temperature and heated to 70 °C for 60 min. The reaction was allowed to continue for 16 h at room temperature. Trimethylsilylation was performed by addition of 15 μl MSTFA + 1% TMCS and incubated for 1 h at room temperature. Finally, 15 μl heptane, including methyl stearate (15 ng/μl), was added as an injection standard.^17^

#### Metabolite analysis

The collected samples were subjected to constrained randomisation within the analytical batches.^18^ The run order of the longitudinal samples was randomised, but consequently run in the same batch and directly adjacent to each other in the analytical run, thereby minimising variability in platform performance for individual patients. The metabolites were analysed with a Leco Pegasus HT time-of-flight mass spectrometer equipped with an Agilent 7890 A gas chromatograph. Leco ChromaTOF software was used for instrument control and raw data acquisition. The column used for the chromatographic separation was a 30 m, 0.25 mm i.d. DB5-MS UI column with 0.25 µm thick stationary phase. Splitless injection of 1 μl sample was performed with a PAL auto sampler system at an injection temperature of 270 °C. The purge time was 75 sec with a rate of 20 ml/min. Helium was used as carrier gas with a flow rate of 1 ml/min. The primary oven temperature was held constant at 70 °C for 2 min and then ramped at 20 °C/min to 320 °C, where it was held constant for 8 min. The transfer line temperature between the gas chromatograph and mass spectrometer was set to 250 °C. Electron impact ionisation at 70 eV was employed with an ion source temperature of 200 °C. Mass spectra were collected in the mass range of *m/z* 50–800 at 20 Hz and 1670 V detector voltage. A series of n-alkanes (C8-C40) were used as external retention index standards. As an additional quality control measure of analytical performance across and within samples batches, we analysed a pooled microdialysis liquid and serum quality control reference sample (QC) at the beginning and end of each analytical batch, as well as between every patient sample group (approximately after every tenth analytical sample). Relative standard deviation percentages (RSD%) were calculated for each detected metabolite.

#### Metabolite identification and quantification

Acquired raw data were exported to MATLAB (Mathworks, Natick, MA) in NetCDF format and processed using the RDA curve resolution script, developed in house. The RDA procedure generates chromatographic profiles for each compound in each sample with a corresponding common spectral profile. We used the integrated area under the resolved chromatographic profile for quantification. The identities of the resolved peaks were determined by comparing mass spectra and retention indices with data in the Swedish Metabolomics Centre in-house spectral library. NIST MS search software was used for manual verification of spectral identification. Compounds with a NIST match score value below 700 and retention index (RI) deviation larger than 25 units from the reference value were excluded. For identification with high confidence, all major fragment ions in the library hit should be present in the resolved spectra with correct spectral intensity profile. Only identified peaks were included in the multivariate analysis. Monosaccharides were defined as a mix of stereoisomers based on their retention index. Some monosaccharide mixes are detected chromatographically as multiple peaks. For example, ketohexoses, including D-Fructose, are found as two peaks due to their rapid transformation into tautomeric pyranose and furanose forms. D-Fructose appears mainly as β-D-fructopyranose and β-D-fructofuranose, approximately in a three to one ratio (in aqueous solution at 20 °C).^19^ The median RSD% for all identified metabolites in serum and dialysis fluid was 7.2 and over 95% of the identified metabolites had an RSD% below 30 (Supplementary Table 1 and 2).

#### Special reagents

All chemicals were of analytical grade. The isotopically labelled internal standards (IS) myristic acid-1,2,3-^13^C_3_, salicylic acid-d_6_ and sucrose-^13^C_12_ were purchased from Cambridge Isotope Laboratories (Andover, MA, USA), Icon (Summit, NJ, USA) and Campro Scientific (Veenendaal, The Netherlands), respectively. The stock solutions for IS were prepared in 0.5 μg/μl concentrations in methanol or water prior to metabolite extraction. Silylation grade pyridine and N-Methyl-N-trimethylsilyltrifluoroacetamide with 1% trimethylchlorosilane were purchased from Restek (Bellefonte, PA, USA).

### Multivariate statistical analysis

Multivariate statistical data analysis was applied to elucidate metabolites and metabolic pattern in the collected metabolite data relating to effect of treatment, differences between short- and long-time survivors as well as differences between microdialysis fluids from tumour and BAT. Two different but related methods were used; orthogonal projections to latent structures discriminant analysis (OPLS-DA)^20^ and orthogonal projections to latent structures effect projections (OPLS-EP).^18^ We used OPLS-DA for independent statistical analysis, short-time survivors vs. long-time survivors and tumour vs. BAT. While OPLS-EP was used to analyse the effect of treatments in dependent samples (pre-treatment vs. day 3 or 6). For all OPLS-DA models, the variables were mean-centred and scaled by division by the pooled standard deviation prior to modelling. For all OPLS-EP models, the variables were only scaled by division by the standard deviation prior to modelling. The significance of the OPLS models was calculated using cross-validated analysis of variance (CV-ANOVA)^21^ and expressed as CV-ANOVA *p*-values.^22^ The number of cross-validation groups equalled the number of patients included in each comparison, i.e. samples originating from the same patient were kept together in the cross-validation.

Statistical significance of metabolites was calculated using Student’s *t*-test; dependent for the OPLS-EP models and independent OPLS-DA models. Correction for multiple testing was done using Benjamini-Hochberg false discovery rate.^23^ A milder criterion for correction of multiple testing was also included to find metabolites of importance when the data were divided into long- and short-time survivors. Here we calculated the distribution of *t*-values in each comparison using bootstrapping. For each variable the average and standard deviation were calculated. Based on the average and the standard deviation, a distribution for each variables *t*-values was calculated. All variables *t*-value distributions were added together forming a probability density function for the t-values in the comparison. In order to get a reference, the same procedure was performed 1000 times using data tables of the same size as the metabolite data but with random numbers form a normal distribution (average = 0, standard deviation = 1). The average of the 1000 probability density functions was used as reference, and *t*-values with ten times higher probability in the comparison than in the references were considered significant.

## Results

### Baseline characteristics

Table 1 lists patient characteristics, microdialysis catheters, treatment time and dose. The median survival after cisplatin administration was 111.5 (10–492) days. Patient no. 7 died 188 days after treatment from a non-disease related cause. In our metabolomic analysis, we identified 113 metabolites in microdialysate from tumour and BAT, and 135 metabolites in corresponding serum samples (Supplementary Tables 1 and 2). OPLS-DA analysis revealed a significant difference in the overall metabolite concentrations in tumour tissue and BAT before cisplatin treatment (*p* = 2.98E-05) (Fig. 1d). After correcting for multiple testing using the Benjamini-Hochberg false discovery rate (FDR) procedure (FDR < 0.05), 45 metabolites showed significant different expression profiles in tumour tissue microdialysate compared to BAT (Table 2). The most obvious difference between tumour and BAT was the significantly elevated tumour levels of a broad range of both proteinogenic and non-proteinogenic amino acids and degradation products of amino acids. In particular, we found high tumour concentrations of glycine, alanine, cyanoalanine, proline and branched-chain amino acids (leucine, isoleucine and valine) and metabolic end-products, uric acid and lactic acid. The most significantly reduced metabolite groups in tumour were the five carbon monosaccharides and related five carbon sugar alcohols. Ascorbic acid, myo-inositol and erythritol were also lower. The levels of the purine nucleosides, inosine and guanosine, and metabolites used for their synthesis, ribose and hypoxanthine, were also highly reduced in tumour compared to BAT. A high concentration of the 2-hydroxy glutaric acid - a known oncometabolite highly expressed in isocitrate dehydrogenase 1/2 mutated cells - was clearly detected in microdialysis fluids from the extracellular compartment of brain tumours from patient number three and ten (Fig. 1e). In these patients, elevated levels of 2-hydroxy glutaric acid was observed upon cisplatin treatment, most likely due to release of intracellular metabolites. By analysing tumour material from earlier resections, tumours from patient three and ten were confirmed to contain IDH1 mutated (R132H) cells while IDH mutations were not found in other patients.

**Table 1:** Patient characteristics and overview of microdialysis catheters, drug delivery and survival after treatment with cisplatin.

Pat.no.	Gender (M/F)	Age (y)	Diagnosis	Treatment catheter(s)	BAT catheter	Days of treatment	Cisplatin dose after 3 days (mg)	Mean cisplatin dose (mg/day)	Total cisplatin dose (mg)	Survival (days)	Long-time surv.
1	F	52	GBM	1 × 100 kDa/40 mm	1 × 100kDA	8.5	5.96	1.95	16.56	153	Yes
2	M	48	GBM	3 × 100 kDa/40 mm	–	5–6	4.44	1.76	10.59	34	
3	M	46	GBM^a^	3 × 100 kDa/40 mm	–	5.5–7	7.04	2.78	17.46	72	
4	M	50	GBM	2 × 20 kDa/30 mm	1 × 20 kDa/30 mm	7–11	2.67	1.06	9.50	492	Yes
5	M	44	GBM	3 × 20 kDa/10 mm	1 × 20 kDa/10 mm	7–11	5.33	2.11	19.00	179	Yes
6	F	72	GBM	3 × 20 kDA/30 mm	1 × 20 kDa/30 mm	9–10	9.16	3.94	36.76	30	
7	F	60	GBM	3 × 20 kDa/30 mm	1 × 20 kDa/30 mm	9–10	8.43	3.49	32.56	188^b^	Yes
8	M	80	GBM	2 × 20 kDA/30 mm	1 × 20 kDa/30 mm	4.5–5.5	5.57	2.20	10.88	10	
9	F	51	GBM	3 × 20 kDa/30 mm	1 × 20 kDa/30 mm	3–4	8.00	3.69	12.28	47	
10	F	40	Oligoastro III^a^	2 × 20 kDa/10 mm	1 × 20 kDa/10 mm	4–5.5	1.00	0.30	1.23	383	Yes

^a^IDH1 mutated

^b^Non-disease related death

**Table 2:** Summary of identified metabolites with significantly different concentrations in microdialysis fluids from tumour compared to BAT, one day prior to cisplatin treatment.

	*p*-value^a^	Fold difference tumour/BAT^b^
Metabolites higher ↑ in tumour vs. BAT, pre-treatment		
Glycine	**0.00032**	7.81
beta-Cyano-L-Alanine	**0.00014**	4.88
Alanine	**0.00043**	4.49
Proline	**0.00012**	4.41
Histidine	0.016	4.39
Proline [+CO2]	**0.0011**	4.31
Uric acid	0.049	4.12
Ornithine	**0.00074**	3.66
Asparagine	**0.00070**	3.08
Lysine	**0.0025**	3.05
2-Aminobutyric acid	**0.000031**	2.85
Valine	**0.00033**	2.80
Aminomalonic acid	**0.00070**	2.76
Tryptophan	**0.0130**	2.75
Asparagine [-H2O]	**0.00063**	2.74
2-oxoisocaproic acid	**0.0024**	2.73
Arginine	**0.0022**	2.67
Tyrosine	**0.00045**	2.60
Ornithine-1,5-lactam (3-Amino-2-piperidone)	**0.0055**	2.44
Leucine	**0.00053**	2.42
Phenylalanine	**0.00090**	2.34
Threonine	**0.0024**	2.31
Glucoheptonic acid	0.037	2.27
Isoleucine	**0.0011**	2.27
Citrulline (Ornthine)	**0.0021**	2.22
5,6-Dihydrouracil	**0.0049**	1.92
3-hydroxybutyric acid	**0.0028**	1.79
Serine	0.035	1.61
Beta-alanine	**0.0050**	1.56
2-Hydroxy-3-methylbutyric acid	0.020	1.41
Nonanoic acid	**0.0043**	1.36
Lactic acid	0.049	1.32
Metabolites lower ↓ in tumour vs. BAT, pre-treatment		
5-carbon sugar alcohol mix (XYLITOL/ARABITOL/RIBITOL)	**0.000041**	0.20
N-acetyl-L-aspartic acid	**0.0017**	0.21
Aldopentose mix 1 (XYLOSE/LYXOSE/ARABINOSE/RIBOSE)	**0.000081**	0.26
Erythritol	**0.00093**	0.37
Guanosine	**0.0014**	0.39
Hypoxanthine	0.041	0.40
Inositol, myo	0.023	0.41
Inosine	**0.0014**	0.43
Ascorbic acid	**0.0041**	0.44
Ketohexose mix 1 (FRUCTOSE/SORBOSE/TAGATOSE/PSICOSE)	0.046	0.47
N-acetyl-mannosamine	0.023	0.54
Glycero-gulo-heptose	0.025	0.68
Octadecenoic acid, 9-E	**0.015**	0.69

^a^Two-tailed Student’s *t*-test. All listed *p*-values in **bold** meet the FDR < 0.05 criteria by the Benjamini-Hochberg procedure

^b^Fold difference ratio before treatment was calculated by dividing average metabolite concentration in tumour with average concentration in BAT

### Early treatment effects

Using OPLS-EP^18^ to compare the metabolite concentrations before treatment with the concentrations after 3 or 6 days of cisplatin treatment, we found significant changes in both tumour tissue and serum (Figs. 1f, h, i). In tumour, the main findings were highly significant increases in glutamic acid, phosphate and spermidine (Table 3). We also observed a significant increase in a broad range of amino acids. In addition, we detected a strong reduction of cysteine as well as decreased levels of monosaccharides, citric and ascorbic acid. In serum, we also observed a significant increase in a broad range of amino acids (Table 3). Here the metabolic shift was more significantly visible in samples taken six days after treatment, indicating a delay between the treatment site and the blood stream (Table 3, Fig. 1h, i). As in tumour tissue, serum had elevated levels of branched-chain amino acids as well as lysine, beta-alanine and phosphate. In addition, the serum samples contained significantly elevated levels of urea following cisplatin treatment, which was not observed in tumour tissue. We also observed overall altered metabolite levels in BAT after 3 days of treatment (Fig. 1g). However, these changes were small in comparison to the changes observed in models for tumour tissue and serum and no individual metabolite reached the predefined significance level.

**Table 3:** Metabolites in microdialysate from tumour tissue (upper table) and serum (lower table) that changed significantly during intratumoural cisplatin treatment of high-grade glioma for 3 and 6 days, in comparison to pre-treatment.

	Day 3		Day 6	
Increasing ↑ with treatment	*p*-value^a^	Fold change^b^	*p*-value^a^	Fold change^b^
Metabolites in microdialysate				
**Alanine**	**0.0050**	1.61	0.10	1.43
**Asparagine**	**0.0089**	1.53	0.59	1.13
**Beta-alanine**	**0.0052**	1.45	0.046	1.21
**Ethanolamine**	0.013	2.28	**0.0016**	2.26
**Glutamic acid**	**0.0060**	11.19	**0.0039**	9.46
**Glycero-gulo-heptose**	**0.0081**	1.48	0.10	1.25
**Glycine**	**0.0060**	1.61	0.031	1.39
**Isoleucine**	**0.00034**	1.82	0.028	1.74
**Leucine**	**0.00081**	1.68	0.026	1.62
**Lysine**	**0.0053**	1.43	0.14	1.36
**Ornithine**	**0.00006**	2.41	**0.0025**	2.61
**Phosphate**	**0.0026**	4.34	**0.00003**	4.11
**Proline**	**0.0029**	1.86	0.015	1.56
**Putrescine**	**0.00092**	1.40	**0.0012**	1.48
**Serine**	**0.0051**	1.64	0.076	1.48
**Spermidine**	**0.00052**	11.00	**0.0036**	3.78
**Threonine**	**0.00081**	1.75	0.043	1.55
**Uracil**	**0.0028**	2.84	0.013	2.43
**Valine**	**0.00092**	1.58	0.052	1.37
Decreasing ↓ with treatment				
**Aldohexose mix (Glucose/Galactose /Mannose/Idose/Altrose)**	**0.0094**	0.67	0.0066	0.52
**Aldopentose mix (Xylose/Lyxose/Arabinose /Ribose)**	**0.0062**	0.43	0.012	0.36
**Ascorbic acid**	0.014	0.51	**0.0026**	0.46
**Citric acid**	**0.0068**	0.59	0.049	0.59
**Cysteine**	**0.00007**	0.16	**0.00011**	0.13
**Gluconic acid-1,5-lactone**	0.016	0.70	**0.002**	0.53
**Isocitric acid**	**0.0070**	0.58	0.032	0.60
**Ketohexose mix 1 (Fructose/Sorbose /Tagatose/Psicose)**^c^	**0.0064**	0.45	0.030	0.46
**Ketohexose mix 2 (Fructose/Sorbose /Tagatose/Psicose)**^c^	**0.0085**	0.45	0.047	0.48
*Metabolites in serum*				
Increasing ↑ with treatment				
**2,3-Dihydroxybutanoic acid**	**0.0014**	1.52	0.013	1.56
**Beta-alanine**	0.0037	1.34	**0.00066**	1.40
**Citrulline (Ornithine)**	0.022	1.35	**0.0029**	1.34
**Ethanolamine**	0.0041	1.26	**0.0020**	1.30
**Isoleucine**	0.043	1.23	**0.0025**	1.34
**Leucine**	0.018	1.17	**0.0025**	1.26
**Lysine**	0.0082	1.31	**0.0022**	1.38
**Malic acid**	0.076	1.17	**0.0024**	1.29
**Phosphate**	0.033	1.13	**0.00046**	1.21
**Pipecolic acid**	**0.00038**	1.44	0.0052	1.58
**Urea**	0.030	1.24	**0.00089**	1.30
**Valine**	0.0081	1.30	**0.00064**	1.34
Decreasing ↓ with treatment				
**Aldohexose mix (Galactose/Gulose/Allose)**	**0.00093**	0.74	0.0048	0.80
**N-acetyl mannosamine**	**0.00064**	0.68	0.037	0.78

^a^Paired two-tailed Student’s *t*-test. All listed *p*-values in **bold** meet the FDR < 0.05 criteria by the Benjamini-Hochberg procedure

^b^Fold change during treatment was calculated by dividing average metabolite concentration after 3 or 6 days of treatment with pre-treatment concentration

^c^Metabolite mix eluting as two separate peaks with Kovats retention indices 1856 and 1866, see methods section

### Treatment responsive markers and survival

**Fig. 2:**
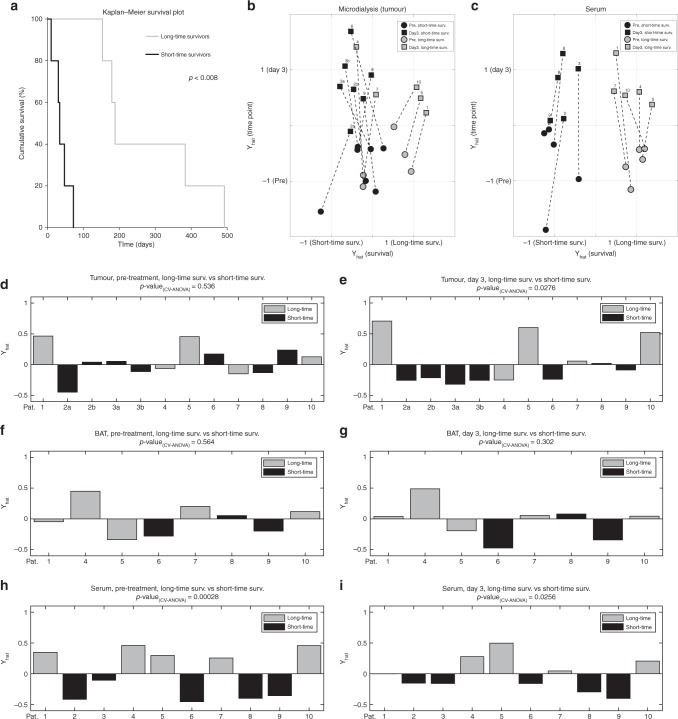
Visualisation of survival and treatment response for long-time and short-time survivors. **a** Kaplan-Meier survival plot for study participants divided into long-time survivors and short-time survivors. Statistical difference indicated with p-value using two-tailed *t*-test. **b**, **c** Scatter plots showing simultaneous modelling of long- vs. short-time survivors and pre-treatment vs. after 3 days of treatment, based on all identified metabolites, in tumour (**b**) and serum (**c**) using OPLS. Axis show estimate of survival-time (*x*-axis) and time point (*y*-axis). Data labels indicate: patient number 1 to 10, short-time survivors in black, long-time survivors in grey, pre-treatment with circle markers and three days of cisplatin treatment with square markers. Samples from the same patient are connected with a dashed line. **d**–**i** Cross-validated multivariate statistical OPLS-DA models comparing metabolite levels detected in long-time survivors vs. short-time survivors: In microdialysis fluids from tumour prior to treatment (**d**) and after three days of treatment (**e**). In microdialysis fluids from BAT prior to treatment (**f**) and after three days of treatment (**g**). In serum prior to treatment (**h**) and after three days of treatment (**i**). Statistical significances for the cross-validated models are indicated by CV-ANOVA.

After completion of cisplatin administration, five patients in the study group had notably longer survival considering the short-expected survival of the included patients. These patients were classified as long-time survivors with a median survival of 188 (153–492) days following treatment. The patients with poor outcome after treatment were classified as short-time survivors with a median survival of 34 (10–72) days (Fig. 2a, Table 1). The differences between long- and short-time survivors could not be linked to age, gender or total dose of cisplatin. It is important to highlight that the relative limited patient number will restricts statistical power and the potential to discover novel response markers. However, simultaneous modelling of survival (long- vs. short-time survivors) and time point (before treatment vs. after 3 days) based on all identified metabolites using OPLS-DA with two responses (survival and time point), showed significant response models for both tumour (*p* = 0.0274) and serum (*p* = 0.00458) (Fig. 2b, c). Scatter plots showing the model estimate for both responses illustrate clearly the metabolic differences between timepoints in both tumour and serum. In serum the differences between short- and long-time survivors was visible at both timepoints (Fig. 2c). In tumour, metabolic differences between long- and short-time survivors were weaker, especially before treatment (Fig. 2b).

**Fig. 3:**
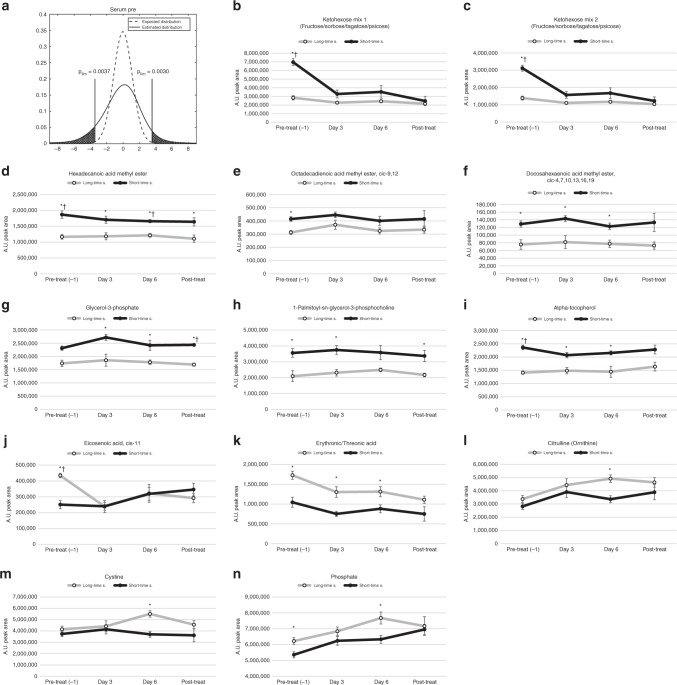
Treatment response patterns for selected metabolites in serum of long-time and short-time survivors. **a** The dashed line indicates the expected t-value distribution if there were no significant differences. The solid line indicates estimated *t*-value distribution from comparisons of metabolite levels in short-time survivor vs. long-time survivor detected in serum prior to treatment. The limits indicate regions where the probability is more than 10 time higher in the estimated distribution. **b**–**q** Metabolites detected in serum with suggestively different concentrations when comparing long-time survivors (○) to short-time survivors (●). Values on *x*-axes indicated sampling times; one day prior to treatment, after 1–6 days of treatment and after the treatment has ended. Values on *y*-axes show mean arbitrary units for quantified peak area ± SEM. *p < 0.05, ^†^**b**–**h** FDR < 0.1.

**Fig. 4:**
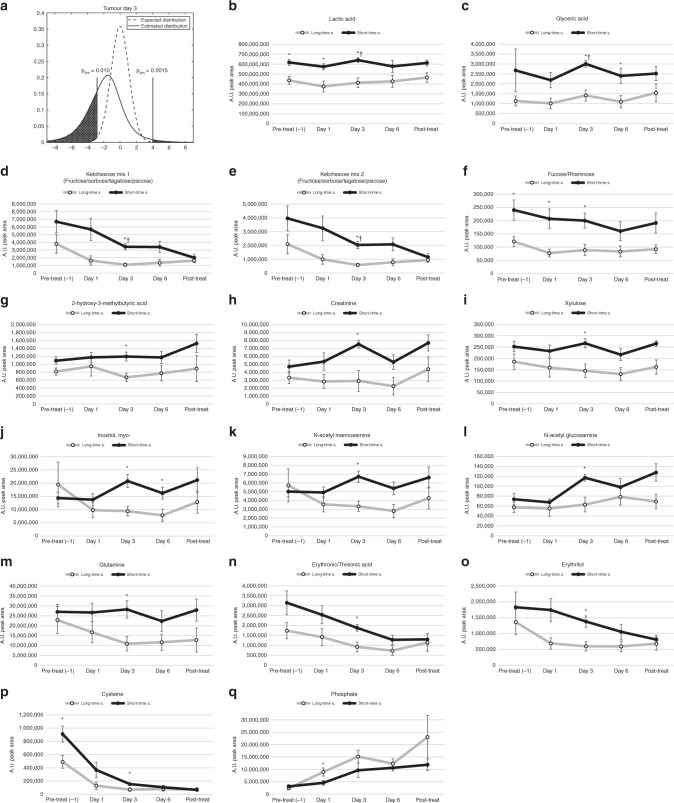
Treatment response patterns for selected metabolites in microdialysate from tumour of long-time and short-time survivors. **a** The dashed line indicates the expected *t*-value distribution if there were no significant differences. The solid line indicates estimated *t*-value distribution from comparisons of metabolite levels in short-time survivor vs. long-time survivor detected in microdialysis fluids from tumours after 3 days of treatment. The limits indicate regions where the probability is more than 10 time higher in the estimated distribution. **b**–**q** Metabolites detected in microdialysis fluids with suggestively different concentrations when comparing long-time survivors (○) to short-time survivors (●). Values on *x*-axes indicated sampling times; one day prior to treatment, after 1–6 days of treatment and after the treatment has ended. Values on *y*-axes show mean arbitrary units for quantified peak area ± SEM. *p < 0.05, ^†^**b**–**h** FDR < 0.1.

To investigate potential markers for therapeutic response, we compared the changes in metabolite concentrations for long- vs. short-time survivors before treatment and after three days of treatment using multivariate OPLS-DA analysis. We observed no significant model separation for long- vs. short-time survivors in tumour or BAT before treatment (Fig. 2d, f) nor in BAT after 3 days of treatment (Fig. 2g). However, the metabolic profiles in tumour and serum were significantly different for long- vs. short-time survivors after three days of cisplatin treatment, with significant cross-validated *p*-values of 0.0276 (Fig. 2e) and *p* = 0.0256 (Fig. 2i), respectively. Interestingly, the largest differences between long- vs. short-term survivors were seen in the metabolic serum profile before treatment (*p* = 0.00028) (Fig. 2h). Since the data were divided into two subgroups, we used a milder penalty for multiple comparisons than FDR to dig deeper into this finding and to learn which individual metabolites that were mainly responsible for the model separations (for description see Material and methods; for visualisation see Figs. 3a and 4a). The resulting metabolites, mainly responsible for separating long-time survivors from short-time survivors identified in serum and tumour microdialysis fluid, are shown in Figs. 3b–n and 4b–q. In serum of long-time survivors, the most striking differences were lower concentration of ketohexoses, mainly fructose, fatty acid methyl esters, glycerol-3-phosphate and the related 1-palmitoyl-sn-glycero-3-phosphocholine, and alpha-tocopherol (Fig. 3b–i). We found a handful of metabolites to be higher in patients with longer survival (Fig. 3j–n). Of these, cis-11-eicosenoic acid, erythronic/threonic acid and phosphate showed enhanced treatment responsive patterns.

We observed a more one-sided distribution of metabolites in microdialysis fluids from tumour. Almost all altered metabolites were lower in long-time survivors (Fig. 4b–p). Levels of tumour-released lactic acid, glyceric acid, ketohexoses and the related deoxy sugar fucose/rhamnose, were significantly higher in patients with short-time survival and were among the metabolites with the most significant changes when compared to patients with long-time survival (Fig. 4b–i). Levels of myo-inositol, N-acetyl mannosamine, glutamine, erythronic/threonic acid and its related alcohol, erythriol, showed a clear response to treatment with a drop-in concentration that was more evident in long-time survivors (Fig. 4g–o). The concentration of cysteine was lower in long-time survivors, but strongly reduced in all patients upon cisplatin treatment (Fig. 4p). Phosphate was the only metabolite in tumour that was found to be higher in long-time survivors (Fig. 4q) and showed a clear treatment responsive increase in both tumour and serum.

## Discussion

Although in vitro experiments have found cisplatin to be effective on glioma cells, cisplatin’s limited ability to penetrate the blood–brain and blood–tumour barriers have restricted its practical use for the treatment of glioblastoma. Therefore, recent focus has been to deliver the drug interstitially^24,25^ or by using targeted methods.^26,27^ In the present study, we used microdialysis which has the advantage of delivering the drug locally and simultaneously allow for the monitoring of treatment effects in the target tissue.^12,13^ The results from this early phase study indicates that cisplatin delivery using the microdialysis technique is safe and allows metabolic monitoring of tumour response.

In this study, interstitial administration of cisplatin in high-grade glioma was highly cytotoxic. Following cisplatin treatment, the metabolic events in the tumour tissue were extensive. On the other hand, multivariate statistical models of the data did not show any major changes in BAT following treatment, demonstrating that the cisplatin treatment has the desired localised effect. In tumour tissue, glutamic acid increased, as a sign of cytotoxicity and many amino acids increased due to protein catabolism, serving as substrates for cellular maintenance and energetic currency. We also found increased levels of spermidine, an indicator of disruption of cellular integrity.

The anticancer activity of cisplatin has been attributed mainly to the formation of cisplatin-DNA adducts, which cause various cellular responses, culminating in apoptosis. Cisplatin is activated through hydrolysis, mainly inside the cell because of the low concentration of chloride ions (3 mM) in the cytosol. Hydrolysis of cisplatin in aqueous solution replaces stepwise the chloro- ligands to form cis-[Pt(NH_3_)_2_(H_2_O)Cl]^+^ and cis-[Pt(NH_3_)_2_(H_2_O)_2_]^2+^, which are very labile, and react readily with DNA nucleobases.^28^ However, the activated cisplatin-aqua complex reacts non-discriminately with many biologically important N-donor ligands or carriers of nucleophilic groups, including proteins, amino acids and nucleic acids.^29,30^ Cisplatin is also known to inhibit mitochondrial respiratory chain complexes and deplete the mitochondrial antioxidant defence system resulting in formation of cellular oxidative stress, which explains its toxicity and complex mechanism of action. It is known that cisplatin also reacts with acetate, phosphate and pyrophosphate groups, which in these cases replaces the chloro ligand. The resulting cisplatin chelates have similar reaction rate towards targets as cis-[Pt(NH_3_)_2_(H_2_O)Cl]^+^, but lower rate than the diaqua form.^28,29^ In our study, we saw a robust increase in levels of phosphate in both microdialysis fluid and serum upon intratumoural cisplatin administration. Interestingly, the release of phosphate was stronger in patients with longer survival, which may be an indication of chemosensitivity with higher turnover and reactivity rate of the phosphate bound cisplatin intermediate in these patients. As discussed below, cisplatin also induced several other metabolic events, a finding also noted in previous reports on cisplatin mechanisms.

Cisplatin has been demonstrated to enhance polyamine catabolism in mice kidneys, which is suggested to be a strong mediator of acute kidney injury.^31^ This finding coincides with our finding of a very strong increase of spermidine in tumour tissue following treatment. Spermidine has an important and complex role in cellular integrity.^32^ It synchronises an array of biological processes (such as Ca^2+^-, Na^+^/K^+^-ATPase) thereby involved in maintaining membrane potential and controlling intracellular pH and volume. In addition, spermidine is involved in regulating biological processes, such as neuroprotection and Ca^2+^ influx by glutamatergic N-methyl-d-aspartate receptor, which has been associated with nitric oxide synthase and cGMP/PKG pathway activation and a decrease of the Na^+^/K^+^-ATPase activity in cerebral cortex synaptosomes. Therefore, we may suggest that a similar increase in polyamine catabolism might play a part in the cisplatin-induced cytotoxicity in glioma.

Reactive oxygen species (ROS) have been shown to exert cytotoxic effects on glioma cells especially when combined with depletion of cysteine.^33^ Cysteine is needed for the synthesis of the antioxidant glutathione, which provides protection from the cytotoxic effects of ROS. Cysteine binds metals and has been shown to interact with cisplatin.^34,35^ The reduced concentration of free cysteine in microdialysis fluids detected here is likely a consequence of cisplatin-cysteine binding and accumulation in the tumour area. This depletion of cysteine will probably result in accumulation of ROS, followed by enhanced cell death. Before treatment, reduced levels of compounds with antioxidant properties—, such as erythritol, N-acetyl-L-aspartic acid and ascorbic acid,—were also detected in the extracellular fluid from tumour tissue compared to BAT. In tumour microdialysate, the levels of cysteine, ascorbic acid and citric acid were further depleted upon cisplatin treatment. Although debatable, presence of compounds with antioxidant properties or those that can sequester cisplatin in the tumour microenvironment could have cisplatin-neutralising effects, resulting in a reduced reactivity of the drug.

Other related mechanisms of possible cisplatin-induced cytotoxicity could result from the increased level of glutamic acid. Activations of inotropic glutamate receptors has shown alterations in calcium homeostasis with eventual cell death from ROS damage. In addition, glutamic acid has been shown to inhibit cysteine synthesis that will impair glutathione production and results in accumulation of ROS that cannot be neutralised. The accumulation of ROS will increase Ca^2+^ release from intracellular stores and cause massive ATP depletion, membrane oxidation and necrosis.^36^ ROS has a complex function in that it may also activate caspases, which are known to induce apoptosis.^37^ In gastric tumour cells cisplatin has been demonstrated to induce caspase-1, which in turn predicts apoptosis.^38^

It is important to note that this study included a relatively small number of treated patients. Obviously, the small patient number restricts the statistical power and the potential to discover novel response markers. This problem is especially prominent in the case where the sample data are divided into two subgroups according to survival time. Although these results should be interpreted with caution, the findings are still of great value for further studies using similar treatment approaches. In the analysed material we found significantly higher levels of lactic acid in microdialysis fluids from tumour of patients with shorter survival, further highlighting lactic acid as a marker of tumour aggressiveness. In both long- and short-time survivors, the lactic acid levels stayed constant and were not reduced during cisplatin treatment, as reported in a model of head and neck squamous carcinoma,^39^ indicating that lactic acid is not a metabolic marker for acute cisplatin toxicity in high-grade glioma patients. However, the obtained metabolic patterns described here are not representative of a naïve tumour, since all tumours have recurred from previous treatments. One of the aims of the present study was to investigate putative candidate markers for therapeutic response. However, there is a risk that the differences in metabolite levels possibly only reflect prognostic differences irrespective of treatment. When analysing single metabolites, the finding of high lactic acid in short-time survivors may indicate that these patients may have a more aggressive tumour compared to the long-time survivors. However, multivariate analysis demonstrated a similarity in tissue metabolic spectra before treatment, and the significant difference in metabolic response following treatment strongly suggests that the performed classification was relevant. Subsequently, the results may be of interest in the current discussion of potential markers for treatment response.

In conclusion, this study demonstrates that significant changes in both the tumour and serum metabolome results from intratumoural administration of cisplatin in malignant glioma. An induction of protein catabolism as well as decreased levels of carbohydrates were evident. The detailed metabolomic analysis reveals that the mechanism of cisplatin cytotoxicity is highly complex, a finding supported in many in vitro studies. Metabolic patterns as well as single metabolites in serum such as phosphate may serve as potential markers for response to therapy. These findings stimulate further studies including other treatment modalities.

## Supplementary information

Supplementary material

## Data Availability

Summarised datasets supporting the conclusions of this article are included within the article and as additional supplementary files.
