# Supplementary material for: Metabolic response patterns in brain microdialysis fluids and serum during interstitial cisplatin treatment of high-grade glioma
Source: Br J Cancer. 2019 Dec 10;122(2):221–32. doi: 10.1038/s41416-019-0652-x (PMC7052137; doi:10.1038/s41416-019-0652-x)
Supplement: Supplementary file 1 — Supplementary material [file 41416_2019_652_MOESM1_ESM.pdf]

Supplementary table 1. Metabolites identified in microdialysis fluid collected from high-grade gliomas.

Metabolite	RSD%[a]	NIST match[b]
**CARBOHYDRATES**		
KETOHEXOSE MIX 1 (FRUCTOSE/SORBOSE/TAGATOSE/PSICOSE)	3.8	963
KETOHEXOSE MIX 2 (FRUCTOSE/SORBOSE/TAGATOSE/PSICOSE)	2.9	947
ALDOHEXOSE MIX 1 (GALACTOSE/GULOSE/ALLOSE)	2.0	932
ALDOHEXOSE MIX 2 (GLUCOSE/GALACTOSE/MANNOSE/IDOSE/ALTROSE)	1.9	924
ALDOHEXOSE MIX 3 (GLUCOSE/GALACTOSE/MANNOSE)	1.8	935
ALDOPENTOSE MIX (XYLOSE/LYXOSE/ARABINOSE/RIBOSE)	4.2	923
DISACCHARIDE MIX 1 (CELLOBIOSE/LAMINARIBIOSE/MALTOSE/TURANOSE)	11.5	865
DISACCHARIDE MIX 2 (MALTOSE/LAMINARIBIOSE/TURANOSE/SAKEBIOSE/LACTITOL)	40.6	835
DISACCHARIDE MIX 3 (MALTOSE/LAMINARIBIOSE/TURANOSE/SAKEBIOSE/LACTITOL)	25.8	804
XYLULOSE	10.0	755
ERYTHROSE	24.2	784
FUCOSE/RHAMNOSE	11.1	852
GLYCERO-GULO-HEPTOSE	31.0	732
ISOMALTOSE	51.0	881
LACTOSE/LACTULOSE	28.0	904
PALATINOSE	42.0	667
SUCROSE	17.1	859
TALOFURANOSE, ETHER	79.8	830
TREHALOSE, ALPHA,ALPHA'	70.1	811
**AMINOACIDS**		
ALANINE	4.7	935
ARGININE	18.1	947
ASPARAGINE	13.7	881
BETA-ALANINE	4.5	837
BETA-CYANO-L-ALANINE	18.2	804
CITRULLINE (ORNTHINE)	2.0	864
CREATININE	34.2	935
CYSTATHIONINE	38.0	922
CYSTEINE	15.9	872
CYSTINE	38.9	904
GLUTAMIC ACID	1.0	941
GLUTAMINE	25.7	935
GLYCINE	2.1	948
GUANOSINE	74.5	785
HISTIDINE	17.1	785
HYPOXANTHINE	11.6	921
INOSINE	54.2	788
ISOLEUCINE	2.9	947
LEUCINE	3.1	931
LYSINE	2.0	906

ORNITHINE	2.7	930
PHENYLALANINE	2.3	948
PROLINE	1.5	945
PROLINE [+CO2]	35.7	899
SERINE	2.6	960
THREONINE	1.7	960
TRYPTOPHAN	19.1	927
TYROSINE	2.7	950
VALINE	3.6	954
**ACIDS**		
2-AMINOBUTYRIC ACID	3.8	938
AMINOMALONIC ACID	20.8	851
ASCORBIC ACID	13.8	834
N-ACETYL-L-ASPARTIC ACID	5.6	899
BENZOIC ACID	8.2	870
CITRIC ACID	3.7	939
DEHYDROASCORBIC ACID DIMER	9.7	861
2,3-DIHYDROXYBUTANOIC ACID	9.8	927
3,4-DIHYDROXYPHENYLACETIC ACID	35.9	824
ERYTHRONIC/THREONIC ACID	4.4	937
GALACTURONIC ACID	16.4	740
GALACTURONIC ACID	3.3	715
GLUCOHEPTONIC ACID	51.7	696
GLUCONIC ACID	28.6	784
GLUCURONIC ACID	24.3	668
GLUTARIC ACID	37.1	859
GLUTARIC ACID, 2-HYDROXY-	7.5	927
GLYCERIC ACID	3.2	968
GLYCOLIC ACID	14.2	701
GLUCONIC ACID-1,5-LACTONE	2.4	722
HEXADECANOIC ACID	5.6	813
DL-ALPHA-HYDROXYBUTYRIC ACID	3.8	946
3-HYDROXYBUTYRIC ACID	5.3	923
2-HYDROXY-3-METHYLBUTYRIC ACID, DERIVATIVE	5.6	761
ISOCITRIC ACID	10.7	906
ALPHA-KETOGLUTARIC ACID	6.6	903
LACTIC ACID	3.3	992
MALIC ACID	5.1	943
NONANOIC ACID	5.2	813
OCTADECENOIC ACID, 9-(E)	14.9	801
OCTADECANOIC ACID	3.2	933
2-OXOISOCAPROIC ACID	4.6	882
PYROGLUTAMIC ACID	8.6	953
SHIKIMIC ACID	34.2	745
URIC ACID	16.0	945

**ALCOHOLS**		
5-CARBON SUGAR ALCOHOL MIX (XYLITOL/ARABITOL/RIBITOL)	5.5	935
6-CARBON SUGAR ALCOHOL MIX (MANNITOL/SORBITOL/GALACTITOL)	5.9	942
ERYTHRITOL	3.9	959
1,5-ANHYDRO-D-GLUCITOL	2.2	935
GLYCEROL	3.8	856
IDITOL	15.5	772
MALTITOL	35.9	796
PALATINITOL	57.5	885
THREITOL	7.4	850
**AMINES**		
N-ACETYL GLUCOSAMINE	19.8	849
N-ACETYL MANNOSAMINE	4.7	777
ETHANOLAMINE	10.2	856
GLUTAMINE	19.0	951
O-PHOSPHOETANOLAMINE	45.9	746
SPERMIDINE	11.8	931
**MISCELLANEOUS**		
ACETAMINOPHEN (PARACETAMOL)*	36.2	934
ACETAMINOPHEN GLUCURONIDE (METABOLITE OF PARACETAMOL)*	94.4	891
BUTYLATED HYDROXYTOLUENE, DERIVATIVE	10.8	845
5,6-DIHYDROURACIL	16.4	954
GLYCEROL-3-PHOSPHATE	35.4	819
INOSITOL, CHIRO	8.1	948
INOSITOL, MYO	2.6	946
INOSITOL, SCYLLO	6.7	916
KYNURENINE	15.4	851
PHOSPHATE (PHOSPHATE-FRAGMENT)	6.0	924
PUTRESCINE	8.1	863
URACIL	11.8	876
UREA	7.6	951

[a]RSD%, calculated relative standard deviation percent for the quantified metabolite in QC samples.
[b]NIST match factor, numeric value (1-999) indicating the similarity of the resolved spectrum to the library spectrum.
*pharmaceutical drug

Supplementary table 2. Metabolites identified in serum collected from high-grade gliomas.

Metabolite	RSD%[a]	NIST match[b]
**CARBOHYDRATES**		
ALDOHEXOSE MIX 1 (GALACTOSE/GULOSE/ALLOSE)	0.5	935
ALDOHEXOSE MIX 2 (GLUCOSE/GALACTOSE/MANNOSE/IDOSE/ALTROSE)	2.9	942
ALDOHEXOSE MIX 3 (GLUCOSE/GALACTOSE/MANNOSE/ALTROSE/TALOSE/GULOSE)	3.8	945
ALDOPENTOSE MIX 1 (XYLOSE/LYXOSE)	16.2	900
ALDOPENTOSE MIX 2 (ARABINOSE/RIBOSE)	2.7	899
DISACCHARIDE MIX 1 (CELLOBIOSE/LAMINARIBIOSE/MALTOSE/TURANOSE)	10.3	837
DISACCHARIDE MIX 2 (MALTOSE/LAMINARIBIOSE/TURANOSE/SAKEBIOSE /LACTITOL)	9.4	872
FUCOSE/RHAMNOSE	8.3	824
ISOMALTOSE	4.7	930
KETOHEXOSE MIX 1 (FRUCTOSE/SORBOSE/TAGATOSE/PSICOSE)	4.7	936
KETOHEXOSE MIX 2 (FRUCTOSE/SORBOSE/TAGATOSE/PSICOSE)	4.1	833
LACTOSE	20.3	794
MELIBIOSE	28.8	852
RIBOSE	8.9	841
TALOFURANOSE, ETHER	54.3	832
SAKEBIOSE	27.6	784
SUCROSE	11.1	861
**AMINO ACIDS**		
ALANINE	3.1	918
BETA-ALANINE	14.6	722
ASPARAGINE	4.0	929
CITRULLINE (ORNITHINE)	8.4	907
ARGININE	4.5	921
ASPARAGINE	8.0	820
ASPARAGINE [-H2O]	4.8	867
CREATININE	8.0	952
CYSTATHIONINE	15.1	706
CYSTEINE	4.5	873
CYSTINE	10.3	944
GLUTAMIC ACID	3.2	933
GLUTAMINE	36.5	947
GLYCINE	5.4	946
HISTIDINE	8.1	867
METHYL HISTIDINE	19.2	745
HYPOXANTHINE	6.3	901
INOSINE	9.6	917
ISOLEUCINE	2.3	879
KYNURENINE	15.7	874
LEUCINE	4.2	902
LYSINE	2.6	938
METHIONINE	1.0	829

PHENYLALANINE	1.8	943
ORNITHINE	4.8	925
PROLINE	3.2	926
PROLINE [+CO2]	17.2	875
TRANS-4-HYDROXY-L-PROLINE	4.1	869
SERINE	15.3	960
TAURINE	10.4	921
THREONINE	3.8	966
TRYPTOPHAN	1.6	929
TYROSINE	2.2	953
VALINE	2.1	910
**ACIDS**		
ADIPIC ACID	10.9	721
2-AMINOBUTYRIC ACID	2.7	935
ASPARTIC ACID	29.5	883
DOCOSAPENTAENOIC ACID	5.6	866
DOCOSAHEXAENOIC ACID	5.4	897
DODECANOIC ACID	5.2	806
ERYTHRONIC ACID	4.7	868
FUMARIC ACID	32.2	896
GALACTURONIC ACID	8.7	749
GLYCERIC ACID	2.5	922
HEPTADECENOIC ACID, CIS-10-	8.4	913
HEPTADECANOIC ACID	6.3	919
HEXADECANOIC ACID	5.7	916
HEXANOIC ACID	14.9	785
2,3-DIHYDROXYBUTANOIC ACID (ISOMER 1)	4.7	941
2,3-DIHYDROXYBUTANOIC ACID (ISOMER 2)	3.3	927
DL-ALPHA-HYDROXYBUTYRIC ACID	4.2	955
3-HYDROXYBUTYRIC ACID	3.1	822
4-HYDROXYPHENYLACETIC ACID	18.1	749
2-ETHYL-3-HYDROXYPROPIONIC ACID, DI-	19.8	789
EICOSADIENOIC ACID, CIS-11,14-	15.3	824
EICOSANOIC ACID	24.0	650
EICOSAPENTAENOIC ACID	8.1	831
EICOSATETRAENOIC ACID, 5,8,11,14-(Z,Z,Z,Z,)	5.4	933
EIOCOSENOIC ACID, CIS-11-	14.7	886
GALACTONIC ACID	40.2	862
GLUCONIC ACID	20.3	862
ALPHA-KETOGLUTARIC ACID	3.5	813
LACTIC ACID	1.5	920
MALIC ACID	1.9	919
MYRISTIC ACID	2.7	826
MYRISTOLEIC ACID	2.5	895
NONANOIC ACID	11.4	732

OCTADECANOIC ACID	7.2	943
OCTADECENOIC ACID,-9-(Z)	2.3	921
OCTADECADIENOIC ACID, 9,12-(Z,Z)	5.0	905
2-OXOISOCAPROIC ACID	2.3	900
PENTADECANOIC ACID	9.0	780
PALMITOLEIC ACID	2.2	953
PIPECOLIC ACID	4.1	835
PYROGLUTAMIC ACID	20.5	890
PYROPHOSPHORIC ACID	2.1	923
PYRUVIC ACID	5.6	777
SACCHARIC ACID	16.8	821
THREONIC ACID	3.8	937
URIC ACID	2.9	942
**ALCOHOLS AND STEROLS**		
5-CARBON SUGAR ALCOHOL MIX 1 (ARABITOL/RIBITOL)	5.4	914
5-CARBON SUGAR ALCOHOL MIX 2 (XYLITOL/ARABITOL)	3.4	927
6-CARBON SUGAR ALCOHOL MIX (MANNITOL/SORBITOL/GALACTITOL)	4.0	922
CAMPESTEROL	16.2	926
CHOLESTEROL	0.8	909
ERYTHRITOL	2.5	939
GLYCEROL	5.8	918
GLYCEROL-3-PHOSPHATE	6.4	917
1,5-ANHYDRO-D-GLUCITOL	0.6	928
IDITOL	6.6	787
BETA-SITOSTEROL	10.9	898
GAMMA-TOCOPHEROL	5.3	862
THREITOL	3.8	922
ALPHA-TOCOPHEROL	6.5	904
**AMINES**		
N-ACETYL MANNOSAMINE	7.3	689
ETHANOLAMINE	3.5	919
O-PHOSPHOETANOLAMINE	22.6	843
**MISCELLANEOUS**		
ACETAMINOPHEN (PARACETAMOL)*	7.3	949
ACETAMINOPHEN GLUCURONIDE (METABOLITE OF PARACETAMOL)*	3.8	903
CITRIC ACID	1.5	932
DOCOSAHEXAENOIC ACID METHYL ESTER, 4,7,10,13,16,19-(Z,Z,Z,Z,Z,Z)	15.8	769
GALACTOPYRANOSIDE, 1-O-METHYL-, BETA-D-	5.3	747
GLYCERO-GULO-HEPTOSE	15.8	800
GUANOSINE	47.8	695
HEXADECANOIC ACID METHYL ESTER	5.7	848
BUTYLATED HYDROXYTOLUENE, DERIVATIVE	3.7	799
INOSITOL, CHIRO	3.1	889

INOSITOL, MYO	1.3	922
INOSITOL, SCYLLO	6.0	879
INOSITOL-PHOSPHATE	10.6	893
BETA-MANNOSYLGLYCERATE	5.2	869
1-MONOSTEAROYLGLYCEROL	22.2	896
1-MYRISTOYLGLYCEROL	14.0	877
OCTADECADIENOIC ACID METHYL ESTER, 9,12-(Z,Z)	6.7	813
1-PALMITOYL-SN-GLYCERO-3-PHOSPHOCHOLINE	8.2	868
PHOSPHATE (PHOSPHATE-FRAGMENT)	2.5	952
5,6-DIHYDROURACIL	3.8	953
UREA	4.8	958

[a]RSD%, calculated relative standard deviation percent for the quantified metabolite in QC samples.
[b]NIST match factor, numeric value (1-999) indicating the similarity of the resolved spectrum to the library spectrum.
*pharmaceutical drug
